# Chronic exposure to low dose of bisphenol A impacts on the first round of spermatogenesis *via* SIRT1 modulation

**DOI:** 10.1038/s41598-018-21076-8

**Published:** 2018-02-13

**Authors:** Rosanna Chianese, Andrea Viggiano, Konrad Urbanek, Donato Cappetta, Jacopo Troisi, Marika Scafuro, Maurizio Guida, Grazia Esposito, Loreta Pia Ciuffreda, Francesco Rossi, Liberato Berrino, Silvia Fasano, Riccardo Pierantoni, Antonella De Angelis, Rosaria Meccariello

**Affiliations:** 1 Department of Experimental Medicine, University of Campania “Luigi Vanvitelli”, Via Costantinopoli 16, 80138 Naples, Italy; 2 Department of Medicine, Surgery and Dentistry “Scuola Medica Salernitana”, University of Salerno, Via S. Allende 1, 84081 Baronissi, Italy; 3 Theoreo srl Spin-off Company of the University of Salerno, Salerno, Italy; 4 Department of Movement Sciences and Wellbeing, University of Naples “Parthenope”, Via Medina 40, 80133 Naples, Italy

## Abstract

Spermatogenesis depends on endocrine, autocrine and paracrine communications along the hypothalamus-pituitary-gonad axis. Bisphenol A (BPA), an estrogen-mimic endocrine disrupting chemical, is an environmental contaminant used to manufacture polycarbonate plastics and epoxy resins with toxic effects for male reproduction. Here we investigated whether the chronic exposure to low BPA doses affects spermatogenesis through the modulation of SIRT1, a NAD^+^-dependent deacetylase involved in the progression of spermatogenesis, with outcomes on apoptosis, oxidative stress, metabolism and energy homeostasis. BPA exposure *via* placenta first, and lactation and drinking water later, affected the body weight gain in male offspring at 45 postnatal days and the first round of spermatogenesis, with impairment of blood testis barrier, reactive oxygen species production, DNA damage and decreased expression of SIRT1. The analysis of SIRT1 downstream molecular pathways revealed the increase of acetyl-p53^Lys370^, γH2AX foci, the decrease of oxidative stress defenses and the higher apoptotic rate in the testis of treated animals, with partial rescue at sex maturation. In conclusion, SIRT1 pathways disruption after BPA exposure can have serious consequences on the first round of spermatogenesis.

## Introduction

Spermatogenesis is a complex biological process that requires the self-renewal and the commitment of diploid spermatogonia for proliferation, the meiosis of spermatocytes and the post-meiotic differentiation of haploid spermatids into mature spermatozoa. This process strongly depends on endocrine control through the hypothalamus-pituitary-gonad (HPG) axis and requires intratesticular autocrine and paracrine communications between germ and somatic cells^1–5^. Environment and diet influence spermatogenesis with known consequences on male fertility^6^. In this respect, a variety of natural and synthetic compounds, collectively named endocrine disrupting chemicals (EDCs), mimics or antagonizes endogenous hormones, interferes with hormone synthesis and clearance, and generates adverse health outcomes in mammals^7–9^. Bisphenol A [2,2-bis (4-hydroxyphenyl) propane, BPA], an estrogen-mimic EDC, is an ubiquitous environmental contaminant used as monomer to manufacture polycarbonate plastics and epoxy resins. It is commonly used to produce plastics that line food/drink containers, goods of common use, thermal receipts, and medical devices such as dental sealants. Heat (as in microwaves or dishwashers) and either acidic or basic conditions (as in foods) accelerate the hydrolysis of ester bonds linking BPA monomers leading to the release of BPA in the environment with potential risk of exposure for living beings. BPA is a well-known toxicant for male reproductive physiology in animal models, but data in humans are quite controversial since exposure doses, duration, route and life stage all affect BPA effects on reproductive health^7,8,10^. Recently, the imbalance of HPG axis, autophagy and apoptosis in testis, meiotic abnormality, impairment of blood-testis barrier (BTB), oxidative stress, decreased sperm quality and epigenetic changes have been reported in BPA-treated males^11–17^. Transgenerational effects on male offspring also occur^15,18^. Given this background, the effects of environmental BPA on male fertility have attracted much attention, with particular interest on the exposure to low BPA doses, especially during critical periods of life such as foetal and perinatal development^10^.

Sirtuins are a family of seven (SIRT1-7) NAD^+^-dependent deacetylases, which have different subcellular localizations (cytoplasm, nucleus and mitochondrion), substrates and functions^19^. Evidence point out the sirtuins as regulators of several processes such as aging, apoptosis, oxidative stress response, mitochondrion biogenesis, metabolism and energy homeostasis^20^ in cancer, cardiovascular and neurodegenerative diseases^21–23^. SIRT1 has a recognized role in spermatogenesis. *Sirt1* knockout male mice are infertile due to the down-regulation of the HPG axis^24^. In this model, spermatogenesis arrests before the completion of meiosis, apoptosis of pachytene spermatocytes occurs, somatic Leydig and Sertoli cells share abnormal maturation and testis produces low testosterone levels. In mouse, highest expression of *sirt1* have been observed in mid to late stages of meiosis^25^. Specific ablation of *sirt1* in pre-meiotic cells revealed direct involvement in spermatogenesis as testis size reduces, spermatogenesis delays and fecundity decreases. At cellular level, pre-meiotic differentiation slows down, post-meiotic stages have defective chromatin condensation, spermatozoa are abnormally shaped and exhibit high levels of DNA damage^25^. Lastly, a possible link between BPA exposure and SIRT1 recently emerged, since long-term exposure to low BPA dose reduces histone acetylation in adult rat testis and in parallel increases the expression/availability of SIRT1^26^.

Here, we investigated whether the chronic exposure of rats (from foetal period until the completion of the first round of spermatogenesis) to low BPA doses affects the first round of spermatogenesis modulating SIRT1 expression and its downstream molecular pathways.

## Results

### Body weight and BPA levels in plasma

Mean body weight (bw) at 45 postnatal days (PND, pubertal animals) was 187.80 ± 7.69 g in control group and 212.00 ± 15.10 g in BPA-treated animals; in young adults (60 PND), it was 260.75 ± 14.09 g in control group and 251.40 ± 11.58 g in BPA-treated animals. The ANOVA for repeated measures demonstrated a significant effect for the time (F1,19 = 44; P < 0.01) and for the treatment x time interaction (F1,19 = 6.5; P < 0.05). The Tukey post-hoc test showed a significant difference between the BPA-treated group and the control group at 45 PND but not at 60 PND.

Plasma BPA levels did not display any statistically significant difference between the control and the treatment groups at any time point. The mean cumulative values of the BPA levels were 0.13 ± 0.04 μg/l for the control groups and 0.16 ± 0.04 μg/l for the BPA-treated groups.

### Testis alterations in BPA-exposed rats

**Figure 1:**
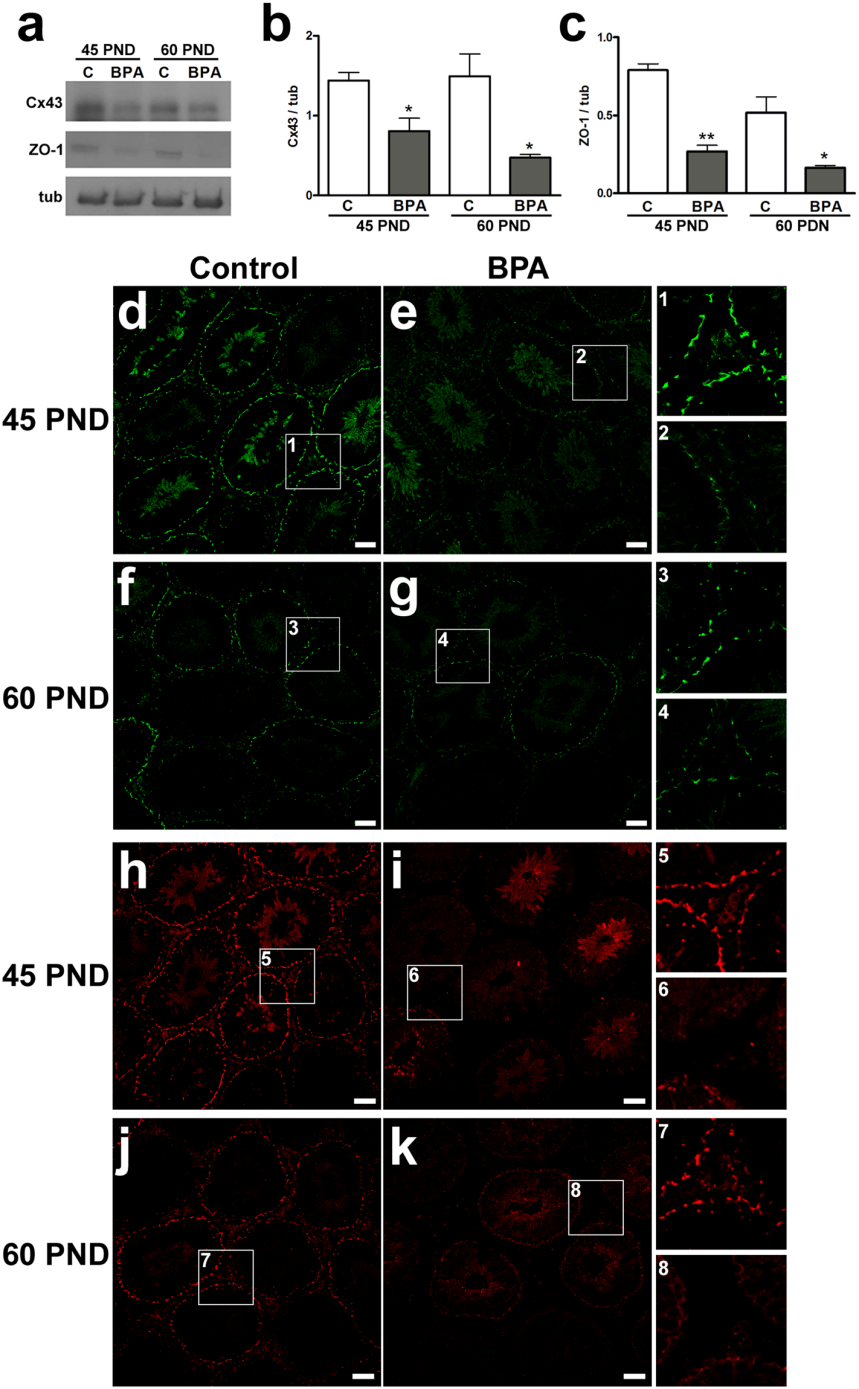
Effects of BPA exposure on integrity of blood testis barrier. (**a**) Western blotting representative bands of Cx43 and ZO-1. (**b,c**) Protein expression of Cx43 (**b**) and ZO-1 (**c**) at 45 PND and 60 PND. Data are expressed as the mean ± SEM. (**d–g**) Immunofluorescent images showing Cx43 (green) localization at 45 PND (**d**,**e**) and 60 PND (**f,g**). (**h–k**) Representative pictures showing distribution of ZO-1 (red) at 45 PND (**h,i**) and 60 PND (**j**,**k**). Areas at higher magnification from each experimental group are depicted in the insets 1–4 (for Cx43) and 5–8 (for ZO-1). *P < 0.05, **P < 0.01 *vs* age-matched control group. Scale bar, 50 µm. Cx43: connexin 43; ZO-1: zonula occludens 1; tub: tubulin; C: control group: BPA: bisphenol A-exposed group; PND: postnatal day.

In order to assess the integrity of seminiferous epithelium that can affect the correct progression of spermatogenesis, we evaluated the expression and the localization of connexin 43 (Cx43) and zonula occludens 1 (ZO-1), well-known markers of the BTB^27^. BPA exposure significantly decreased Cx43 and ZO-1 levels at both 45 and 60 PND compared to control with most significant effects on ZO-1 in 45 PND treated animals (P < 0.01) (Fig. 1a–c). Immunofluorescence analysis for Cx43 and ZO-1 carried out on testis of 45 and 60 PND clearly revealed a strong speckled signal at the junction sites between Sertoli and germ cells in control rats (Fig. 1d,f,h,j). In contrast, Cx43 and ZO-1 immunofluorescence signals were scattered and significantly reduced in BPA-exposed animals. Such an impairment was more pronounced at 45 PND (Fig. 1e,g,i,k).

### DNA damage and oxidative stress in BPA-exposed rats

**Figure 2:**
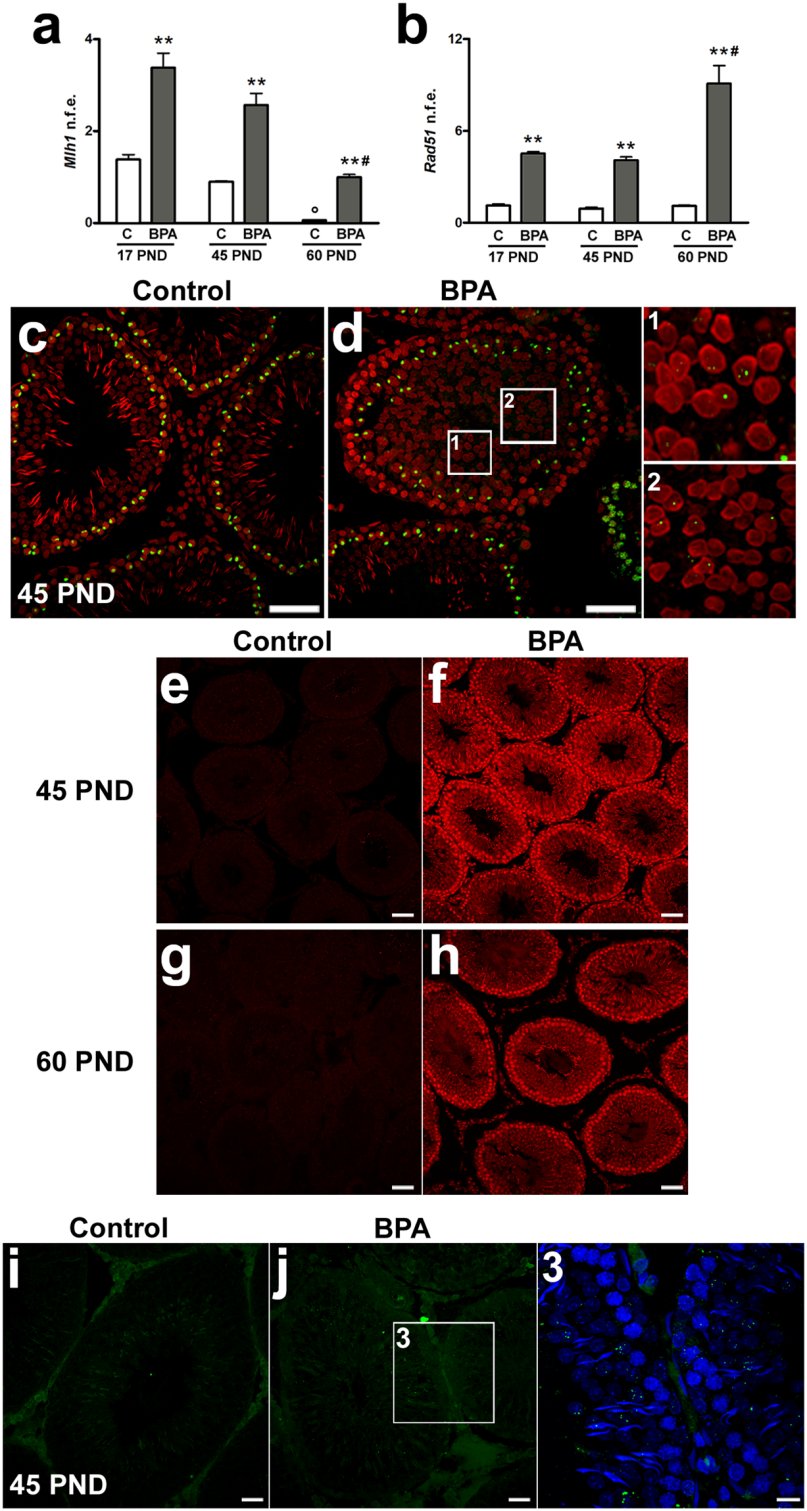
Effects of BPA exposure on oxidative stress and DNA damage. (**a**,**b**) mRNA expression of *Mlh1* (**a**) and *Rad*51 (**b**) at 17, 45 and 60 PND. Data are reported as normalized fold expression (n.f.e.) ±SEM. (**c,d**) Detection of γH2AX (green) by immunofluorescence at 45 PND. Areas at higher magnification are shown in the insets 1–2. Nuclei are labelled with propidium iodide (red). (**e–h**) Generation of oxidants as showed by DHE (red) content in testicular tissue at 45 PND (**e,f**) and 60 PND (**g,h**). (**i,j**) Assessment of oxidative stress-induced DNA damage by 8-OHdG (green) at 45 PND. Area at higher magnification from BPA-exposed group is shown in the inset 3. Nuclei are labelled with DAPI (blue). **P < 0.01 *vs* age-matched control group; P < 0.05 *vs* 17 and 45 PND control groups; ^#^P < 0.01 *vs* 17 and 45 PND BPA groups. Scale bar, 50 µm. DHE: dihydroethidium; 8-OHdG: 8-OH-deoxyguanosine; C: control group: BPA: bisphenol A-exposed group; PND: postnatal day.

Due to the defective expression of proteins in the BTB, the possibility of BPA-induced DNA/tissue damage was examined. In order to evaluate the presence of DNA breaks, the expression rate of the crossover-associated protein *Mlh1*, a marker of DNA mismatch repair system^28,29^, and of *Rad51*, which encodes DNA repair protein, were assayed by quantitative real-time RT-PCR (qPCR). BPA exposure significantly increased *Mlh1* expression compared to control groups (P < 0.01) at each time point. Of note, during postnatal testis development, we observed a time-dependent decrease in *Mlh1* expression in both control and BPA-exposed animals (Fig. 2a). Conversely, a significant increase in *Rad*51, a recombinase positively correlated with resistance to genotoxic treatment^30^, was detected in all BPA-exposed animals (P < 0.01 *vs*. control), but highest expression rates were observed at 60 PND (P < 0.01) (Fig. 2b). The presence of DNA breaks was further confirmed by immunofluorescence for γH2AX, a histone H2A variant phosphorylated on Ser 139^31^. Apart from canonical immunolocalization in spermatocytes (from leptotene to early zygotene and in sex body of pachytene spermatocytes), γH2AX foci also appeared in round spermatids in BPA-treated animals but not in control animals (Fig. 2c,d).

These phenomena could stem from the well-known pro-oxidant properties of BPA. Thus, the production of reactive oxygen species (ROS) and possible oxidative modification of DNA were evaluated by dyhydroethidium (DHE) and immunostaining for 8-hydroxy-2′-deoxyguanosine (8-OHdG). Nuclear DHE labelling was barely detected in the testis of control animals at 45 and 60 PND (Fig. 2e,g). Conversely, strong DHE signal was present in both germinal and interstitial compartment of 45 and 60 PND treated animals (Fig. 2f,h). Immunofluorescence for 8-OHdG revealed diffused DNA damage in the germinal epithelium of treated animals but not in controls (Fig. 2i,j).

### Testicular expression of SIRT1 in BPA-exposed rats

**Figure 3:**
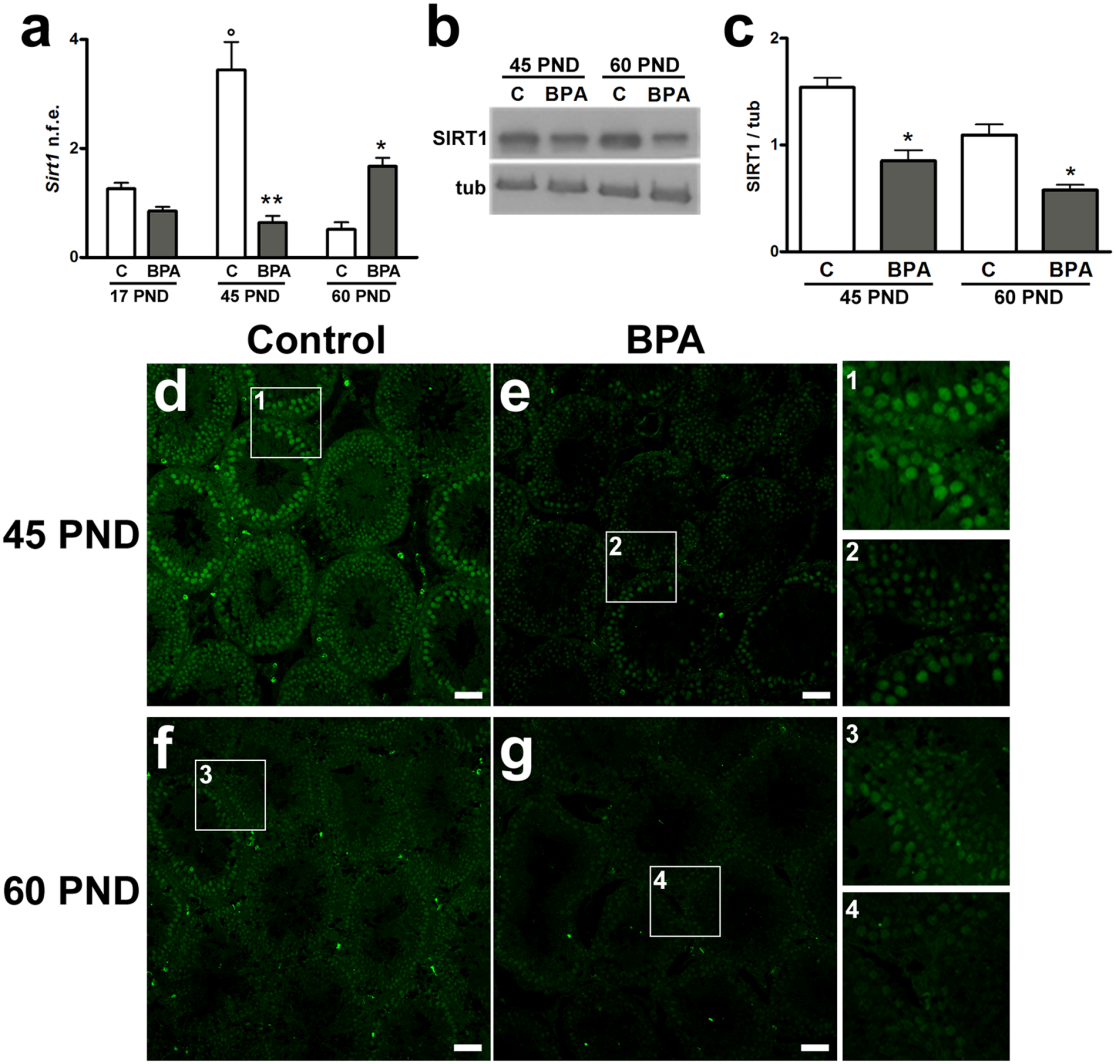
Effects of BPA exposure on testicular SIRT1 expression. (**a**) mRNA expression of *sirt1* at 17, 45 and 60 PND. Data are reported as normalized fold expression (n.f.e.) ±SEM. (**b**) Western blotting representative bands of SIRT1. (**c**) SIRT1 expression level in rat testis at 45 PND and 60 PND. Data are expressed as the mean ± SEM. (**d–g**) Representative images showing SIRT1 (green) at 45 PND (**d**,**e**) and 60 PND (**f**,**g**). Selected areas from each experimental group are magnified in the insets 1–4. *P < 0.05, **P < 0.01 *vs* age-matched control group; °P < 0.01 *vs*17 and 60 PND control groups. Scale bar, 50 µm. tub: tubulin; C: control group: BPA: bisphenol A-exposed group; PND: postnatal day.

Given the essential role played by SIRT1 in cell differentiation and reproductive function in testis^25^, *sirt1* mRNA expression was examined by qPCR during the first round of spermatogenesis at 17, 45 and 60 PND. *Sirt1* levels in control groups significantly changed during the first round of spermatogenesis with highest expression levels observed at 45 PND and lowest expression rate observed at 60 PND (P < 0.01). This particular expression profile did not occur in BPA-treated animals (Fig. 3a). At 17 PND, no significant difference was observed between exposed animals and control group; instead, at 45 PND, *sirt1* expression significantly decreased in BPA-exposed animals (P < 0.01 *vs*. 45 PND control group); at 60 PND, the mean expression levels of *sirt1* were higher in BPA-treated animals (P < 0.05 *vs*. 60 PND control group) (Fig. 3a). Additionally, we examined SIRT1 protein levels by Western blot at 45 and 60 PND revealing a significant decrease of SIRT1 protein at both 45 and 60 PND in BPA-exposed rats compared to control (P < 0.05) (Fig. 3b,c). The localization of SIRT1 was analyzed by immunofluorescence at 45 and 60 PND. Signal was almost homogeneously diffused in tubule, especially at 60 PND, in both control and BPA-exposed animals. Lower signals were observed in BPA-exposed animals at each time points. At 45 PND, a stronger signal was appreciated in mitotic/early meiotic stages and slightly in spermatids. Sertoli and Leydig cells were also immunopositive (Fig. 3d,e). At 60 PND, SIRT1 localization was as similar as at 45 PND (Fig. 3f,g).

### Molecular pathways related to SIRT1 downregulation in BPA-treated animals

**Figure 4:**
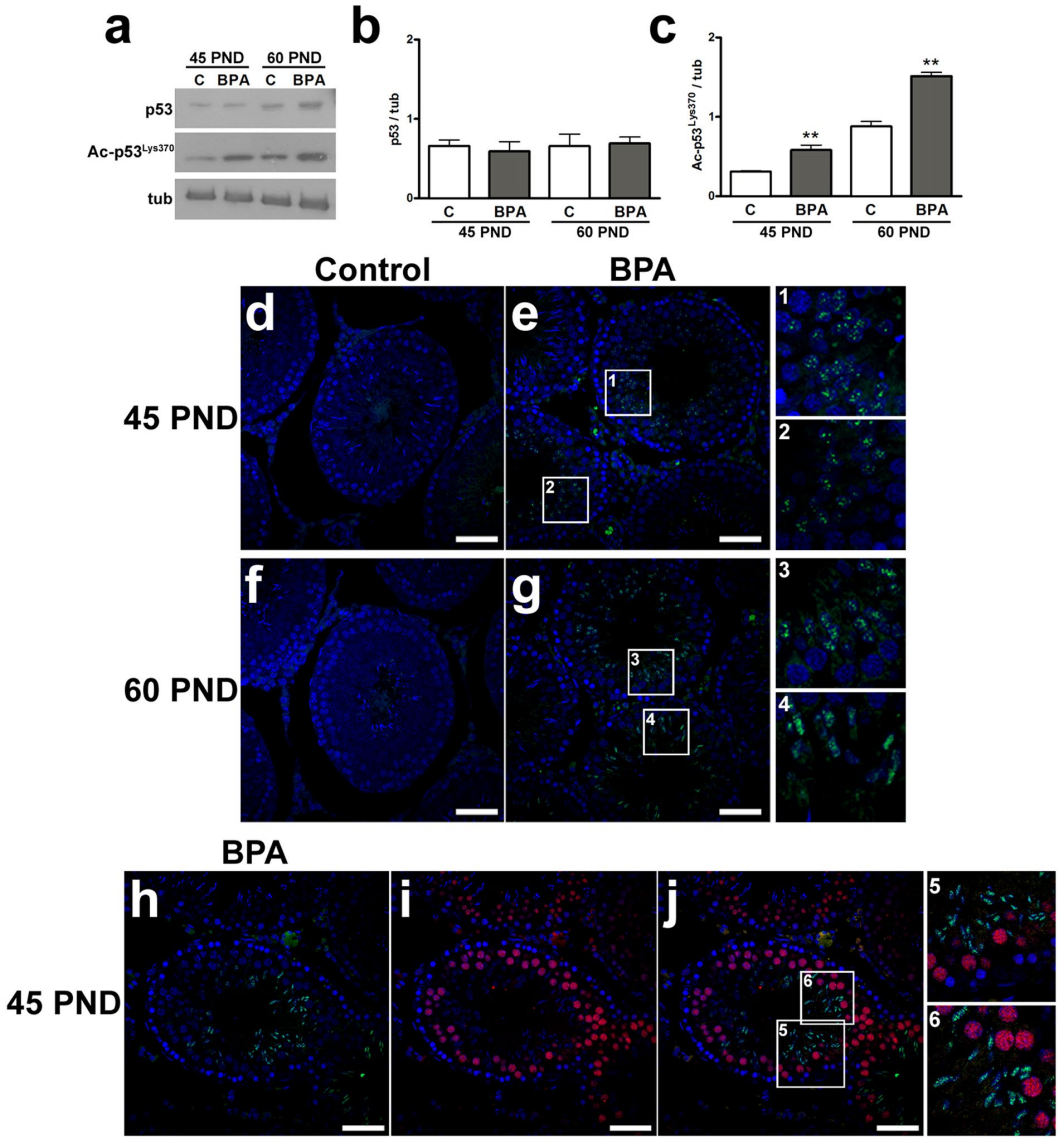
DNA damage response induced by exposure to BPA. (**a**) Western blotting representative bands of p53 and Ac-p53^Lys370^. (**b,c**) Protein expression of p53 (**b**) and Ac-p53^Lys370^ (**c**) at 45 PND and 60 PND. Data are expressed as the mean ± SEM. (**d–g**) Ac-p53^Lys370^ (green) expression in rat testis at 45 PND (**d**,**e**) and 60 PND (**f**,**g**). The areas within the squares are shown at higher magnification in the insets 1–4. (**h–j**) Representative images evaluating the expression of Ac-p53^Lys370^ (green) (**h**), SIRT1 (red) (**i**) and their merge (**j**) at 45 PND from BPA-exposed group. Areas at higher magnification are shown in the insets 5–6. Nuclei are labelled with DAPI (blue). **P < 0.01 *vs* age-matched control group. Scale bar, 50 µm. Ac-p53^Lys370^: acethyl-p53^Lys370^; tub: tubulin; C: control group: BPA: bisphenol A-exposed group; PND: postnatal day.

Consequently, to evaluate one of the principal downstream effector of SIRT1 signaling, the level of p53 acetylation was investigated by Western blot. While the expression levels of total p53 did not differ, BPA exposure significantly increased the levels of acetyl-p53^Lys370^ (Ac-p53^Lys370^) at 45 as well as at 60 PND (P < 0.01) (Fig. 4a–c). Consistently, immunofluorescence analysis revealed the significant appearance of Ac-p53^Lys370^ signal in BPA-exposed animals, at 45 and 60 PND. Ac-p53^Lys370^ was detected in post-meiotic stages, especially in spermatids at acrosome phase (P < 0.01) (Fig. 4d–g). Double immunofluorescence was carried out to study both SIRT1 and Ac-p53^Lys370^. Interestingly, while Ac-p53^Lys370^ was located in post-meiotic stages, SIRT1 was mainly located in meiotic stages (Fig. 4h–j).

### Anti-oxidant defense in BPA-exposed animals

**Figure 5:**
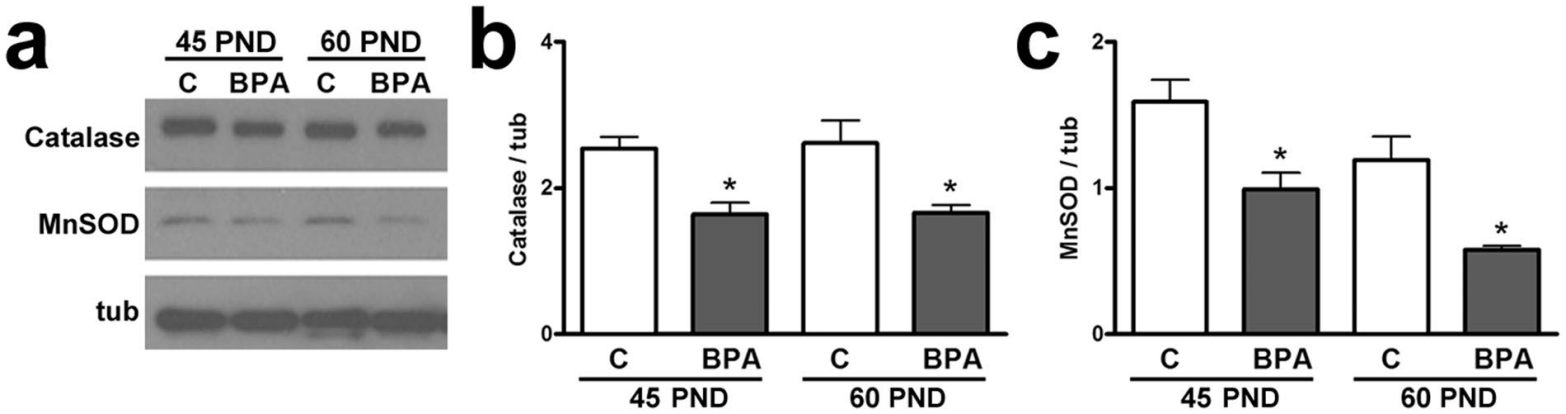
Antioxidant enzymes after exposure to BPA. (**a**) Western blotting representative bands of Catalase and MnSOD. (**b,c**) Protein expression of Catalase (**b**) and MnSOD (**c**) at 45 PND and 60 PND. Data are expressed as the mean ± SEM. *P < 0.05 *vs* age-matched control group. tub: tubulin; C: control group: BPA: bisphenol A-exposed group; PND: postnatal day.

The strict relationship between SIRT1 function and ROS accumulation is well established. At this regard, Western blot analysis for enzymatic defense against the oxidative stress in tissues has been performed. Our findings showed that both catalase and MnSOD significantly decreased at both 45 and 60 PND in BPA-exposed animals, compared to controls (P < 0.05) (Fig. 5a–c).

### Apoptosis in BPA-exposed animal testis

**Figure 6:**
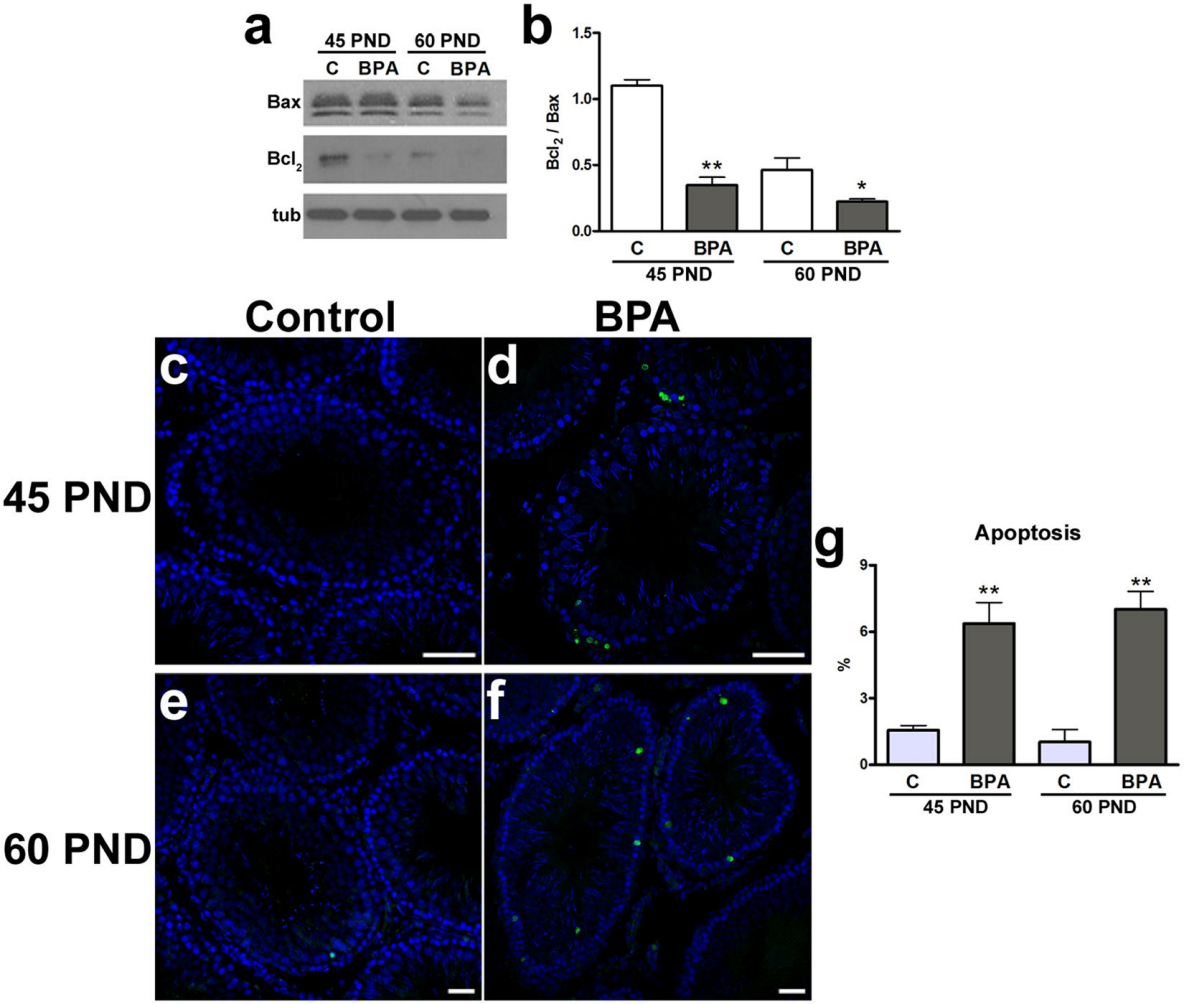
Effects of BPA exposure on apoptosis. (**a**) Western blotting representative bands of Bax and Bcl_2_. (**b**) Protein expression of Bax and Bcl_2_ expressed as ratio at 45 PND and 60 PND. (**c–f**) Apoptotic cells (green) are shown by TUNEL assay in rat testis at 45 PND (**c**,**d**) and 60 PND (**e**,**f**). (**g**) Fraction of tubules with more than three apoptotic cells. Data are expressed as the mean ± SEM. *P < 0.05, **P < 0.01 *vs* age-matched control group. Scale bar, 50 µm. tub: tubulin; TUNEL: terminal deoxynucleotidyltransferase-mediated dUTP nick end labelling; C: control group: BPA: bisphenol A-exposed group; PND: postnatal day.

To better understand the physiological significance of the upregulation of Ac-p53^Lys370^ in testis after BPA exposure, we analyzed protein levels of two typical modulators of apoptosis, Bcl_2_ and Bax. Bcl_2_/Bax ratio were significantly lower in BPA-exposed animals at both 45 (P < 0.01) and 60 PND (P < 0.05) (Fig. 6a,b). Additionally, TUNEL assay was performed to detect apoptotic cells. TUNEL signal was detected in the germinal compartment at basal levels and in Sertoli cells in BPA-exposed animals at 45 and 60 PND (Fig. 6c–f). Compared to respective controls, a significantly higher fraction of apoptotic cells/tubule was revealed in BPA-exposed animals (Fig. 6g).

## Discussion

BPA exposure may have different outcomes on reproductive health depending on age, doses, exposure window and route^7,8,10^. In this study, we evaluated the effects of chronic exposure to the low dose of BPA, from foetal period to sexual maturation, on postnatal testis development. This theme is of particular interest since the exposure to environmental doses of BPA affects reproductive physiology in animal models^7,8,10^, and most importantly, measurable levels of BPA have been detected in urine sample from humans as a consequence of ubiquitous exposure to environmental BPA^32^. The determination of a “safe” BPA exposure level deserves much attention because disparities remain in the tolerable daily intake of BPA being 4 μg/kg bw/day and <50 μg/kg bw/day accordingly to the European Food Safety Agency (ESFA) and the U.S. Environmental Protection Agency (EPA), respectively^33^.

In the present study, 0.1 mg/l BPA was orally administered to dams or weaned offspring *via* drinking water and a daily dose of 10 μg/kg bw was calculated based on daily drinking consumption. Thus, the BPA dose used here was lower than or within the reference limit for humans, currently considered “safe” by ESFA and by EPA. The finding that, despite the constant exposure paradigm used, serum levels of BPA did not differ between exposed and control groups might argue for a contamination problem and points out that any outcome on physiological processes may depend on oral exposure route. This finding is exactly the key-point of the present work, demonstrating that “normal” level of serum BPA cannot assure that there are no ongoing biological effects. A possible explanation for these effects could be the accumulation of BPA in adipose tissue as a consequence of long term oral exposure^34^. Moreover, because the pharmacokinetics of BPA differs in neonatal and adult rats^35^, a differential excretion rate of BPA in pups cannot be excluded.

In the present work, it was found an increased bw in exposed males compared to controls at 45 but not at 60 PND. This finding suggests that the present level of exposure could not cause obesity in the adult but could interfere with the normal temporal pattern of growth. In other words, a low level of BPA could mimic a slight anticipation of puberty. Another possibility, on the other hand, is that the chronic administration of low BPA *via* placenta first, lactation and drinking water later, affected the bw gain in male offspring in pubertal animals only. Consistently, *in vitro* and *in vivo* studies have demonstrated the effects of BPA on adipocyte differentiation, lipid accumulation, glucose transport and adiponectin and that BPA exposure in perinatal period has an obesogenic effect in adulthood with dose and sex dependent outcomes in rodents^36^. In contrast, recent evidence in male mice suggests that BPA exposure reduces bw regulating hypothalamic anorexigenic circuits^37^.

In parallel to the effects on bw, the cytoarchitecture of the seminiferous epithelium was impaired in BPA-exposed animals due to low expression and scattered localization of Cx43 and ZO-1 in BTB. This structure establishes functional Sertoli-germ cells communications^27^ and has been previously accredited as early target for testicular toxicants^38^. The physiology of BTB strongly depends on steroid activity^27^ and thus, aberrant localization of junctional proteins and their reduced amounts are consequences of BPA exposure^16,38,39^.

In testis, like in other organs, junctional microstructure is essential for tissue homeostasis and thus remodelling of these structures may be involved in oxidative stress-induced cell death^27,40^. In our study, massive and diffuse ROS production occurred in the testis of BPA-exposed animals, associated with DNA oxidative damage (8-OHdG) and double strand breaks (γH2AX foci) - especially in germ cells. Therefore, in the presence of BPA, the first round of spermatogenesis suffers from oxidative stress damage. As a consequence, upregulation of *Mlh1* and *Rad51* transcripts, which encode proteins that facilitate crossing over, DNA mismatch repair and recombination^28,29,41^, has been observed in BPA-exposed animals, suggesting meiotic cells as main targets for BPA. Consistently, neonatal exposure to BPA significantly reduces crossover and meiotic recombination permanently reducing sperm production by affecting the pool of spermatogonial stem cell of the developing testis^42^. However, the highest expression rate of *Rad51* in 60 PND treated animals confirms previous data concerning its possible involvement in the resistance to genotoxic damage and the occurrence of genomic instability^30^.

At molecular level, the pro-survival factor SIRT1 that is also a “sensor” of ROS, may be considered one of the key element in the BPA-triggered signaling pathways that, in turn, affect spermatogenesis. Interestingly a similar effect has been reported in *sirt1*^−/−^ mice in which the amount of mature sperm with DNA damage was higher in adult *sirt*^−/−^ mice than in wild type^43^. During postnatal testis development, SIRT1 expression revealed a physiological expression peak in pubertal animals, when spermiogenesis starts, consistently with its functional role in meiotic progression, chromatin condensation and nuclear shaping^25^. In this study, the expression of SIRT1 resulted to be negatively affected by BPA exposure from pubertal period until the completion of the first round of spermatogenesis. SIRT1 impairment can lead to the loss of control of the acetylation of target proteins. Indeed, increased Ac-p53^Lys370^ paralleled with an intra-testicular ROS production, DNA damage and reduced enzymatic defenses against oxidative stress occurred following BPA exposure. Such a situation has been observed until sexual maturation.

The perturbation of Sertoli-germ cells interaction found in the present study may impair the quality of spermatogenesis with consequences on post-meiotic cells. In this respect, the involvement of SIRT1 in the progression of spermatogenesis is well-documented^25,43^ and data here provided account for mitotic, early meiotic and Sertoli cells as the main target cells influenced by SIRT1. Thus, SIRT1 impairment may affect the progression of spermatogenesis towards late meiotic and post-meiotic stages, and the maturation of spermatozoa as well. Consistently, both Ac-p53^Lys370^ and γH2AX foci have been localized in post-meiotic spermatids suggesting the formation of poor quality spermatozoa with possible transgenerational effects on the offspring. Future studies should assess fertility rate during adulthood using the animal model proposed in the present study.

In addition to oxidative stress damage, the testis of exposed animals revealed higher apoptotic rate *vs* control groups. The role of oxidative stress as mediators of apoptosis is well documented in testis, where it leads to poor semen quality, decreased fertilizing capacity, infertility and even adverse pregnancy outcomes^44^. In our experiments, a lowered Bcl_2_/Bax ratio and increased apoptotic rate of pre-meiotic spermatogenesis stages and Sertoli cells were observed. The chronic exposure to low BPA dose not only affected the formation of mature spermatozoa but also influenced the functional microenvironment for proper development of mitotic and post-meiotic cells. This might have a double consequences on spermatogenesis: i) increased apoptosis of pre-meiotic stages located in the basal compartment and Sertoli cells; ii) accumulation of oxidative damage in post-meiotic cells located in the luminal compartment. p53-dependent apoptosis of pre-meiotic stages is one of the major checkpoint to ensure the optimal germ/Sertoli cell ratio and the progression towards meiotic and post-meiotic stages of qualitatively safe spermatocytes. Mid-pachytene spermatocytes, but also spermatogonia, appear poised to undergo apoptosis especially during the first round of spermatogesis^45^, and this process in rat peaks at approximately three weeks of age, being a key step in the development of the sexual maturation and competence. Interestingly, at sexual maturation (60 PND), BPA exposure caused a less pronounced impairment of Cx43/ZO-1 in the BTB and a strong decrease of *Mlh1* mRNA with respect to 45 PND. Taken together, these data suggest a possible rescue of spermatogenesis at later time points. Thus, the first wave of spermatogenesis may be a critical target for BPA. Consistently, intra-peritoneal administration of BPA to pubertal rats leads to oxidative stress and endocrine disorders, which in turn cause apoptosis and authophagy in testis^17^. However, oral administration of environmental levels of BPA for 14 days suppresses reproductive hormones and promotes germ cell apoptosis also in adult rats^12^.

The BPA-dependent modulation of SIRT1 in rat testis has been recently reported^26^. In particular, Chen and co-workers did not observe any effect on bw after 35 weeks of exposure of adult male rats to a “safe” dose of BPA (50 μg/kg bw/day in corn oil), but they reported that SIRT1 dissociated from caveolae, and in turn its up-regulation reduced the acetylation levels of protein substrates. Our study differs as for the age of exposed rats (from foetal to 60 PND instead of adult rats), the dose (10 μg/kg bw/day instead of 50 μg/kg bw/day) and the agent formulation (dissolved in drinking water instead of corn oil). Thus, it clearly emerges that the effects of BPA on reproductive health depend on dose and exposure windows across the lifespan. Here, we expose animals all over their life, from foetal period, throughout lactation, weaning until sexual maturation. The question if the phenotype reported here results from the exposure within a specific timeframe remains to be answered. Since foetal and postnatal germ cell differentiation require SIRT1 activity^25,43^, it can be argued that earlier exposure might be the most dangerous. Consistently, the expression of *Mlh1* in exposed pups (17 PND) was higher than at 60 PND and higher than in the 17 PND control group, providing evidence of early requirement of DNA repair machinery. However, BPA can reach the foetus^46,47^ thus, at present, we cannot exclude transplacentally transfer of BPA with potential damage on gonocytes during gestational stages. Furthermore, BPA injection to neonatal and to late infantile-prepuberal rats impairs fertility and the expression of Sertoli junctional proteins in the BTB^16,39^, consistently with our data. In this respect, the transfer of BPA *via* lactation to newborns during the perinatal period deserves particular attention. Moreover, maternal transfer of BPA during lactation causes sperm impairment in male mice offspring with occurrence of testicular oxidative damage and the total impairment of antioxidant capacity^48^, similarly to data provided here. Interestingly, the milk of BPA-exposed dams has a lipid concentration different from those of the controls^49^, since BPA accumulates in milk and is likely transferred to pups in a much concentrated form^50^, suggesting the great potential of BPA to reach toxic levels in pups^35^.

The aforementioned effects of BPA may also be the consequence of HPG impairment, as previously reported^11,12,51^ or the results of BPA interference in intragonadic steroid signaling, a key step for the correct progression of spermatogenesis^5^.

## Conclusions

Chronic exposure to BPA at doses usually considered safe for health can still have serious consequences on reproductive system. Our data show that chronic exposure at BPA has higher impact on the first round of spermatogenesis than on spermatogenesis progression at sexual maturation, possibly as the consequence of exposure at gestational and neonatal phases. Hence the importance of environmental exposure to ECDs during the early stage of life with particular attention to gestation, lactation and childhood.

## Methods

### Animals and BPA exposure protocol

Six female (200–250 g) and three male (250–300 g) Wistar rats (Harlan Laboratories) were used. Three male-female couples were randomly assorted and housed in the same cage for one week; the males were then coupled with the remaining females for another week. After the coupling period, each female was housed in a separate cage and was given BPA (Sigma-Aldrich), or vehicle (n = 3/group) in the drinking water. BPA was first dissolved in ethanol (100 mg/ml) and diluted 1:100 with ethanol; finally, 0.1 ml of the last solution was added to 1 l of tap water in glass bottles, resulting in 0.1 mg/l BPA. The vehicle consisted of 0.1 ml/l ethanol. Dams received the treatment all over lactation and at weaning; each newborn received the same treatment of the mother *via* drinking water. To analyze the possible effects of BPA on the first round of spermatogenesis, the male newborns were sacrificed at 17 PND (late infantile), 45 PND (pubertal), or 60 PND (young adult), randomly choosing a total of five animals/treatment/time point from different litters. For each animal, one testis was stored at −80 °C and the contralateral was fixed in Bouin’s fluid.

All over the experiments, rats were housed under standard temperature and humidity conditions with a 12:12 light/dark cycle (lights on at 07:00 am) and free access to standard fresh food and water. At sacrifice, animals were deeply anesthetized by overdose of Tanax (0.1 ml intrapulmonary). Experimental protocols were approved by the Ethical Committees of the University of Salerno, and by the Italian Ministry of Education, University and Research (authorization number 45/2014-PR 17/11/2014). All experimental procedures complied with the rules of the European Union Guide for the Care and Use of Laboratory Animals.

### Plasma BPA measure

From anesthetized animals, plasma was collected by centrifugation of whole blood (800 *g* for 15 min). The determination of BPA was conducted as previously reported^52^, with some minor modifications. Briefly, d16-BPA was added as internal standard and samples were defatted with hexane and then extracted with dichloromethane. The extracts were purified in two successive Solid Phase Extraction steps, one with a Florisil solid phase and one with a C18 solid phase. Deconjugation was carried out using β-Glucuronidase/Arylsulfatase from Helix pomatia (Sigma-Aldrich).

Chromatographic separations were carried out using a LCMS-8050 triple quadrupole mass spectrometer equipped with a Nexera UHPLC System (Shimadzu). BPA was separated on an Acquity UPLC BEH (2.1 mm × 50 mm, 1.7μm) C18 column (Waters). A 1.5 min linear gradient was used from 10–95% methanol in water followed by a hold at 95% for 1.0 min at a flow rate of 0.4 ml/min. Negative ion electrospray mass spectrometry with selected reaction monitoring (SRM) and a dwell time of 50 ms per transition was used for the measurement of each analyte. The SRM transitions for BPA were m/z 227.20 to 212.20 (quantifier) and m/z 227.20 to133.15 (qualifier).

### Total RNA extraction, cDNA preparation and qPCR

Total RNA was extracted from rat testis (n = 5) using Trizol reagent (Life Technologies) following the manufacturer’s instructions. Genomic DNA contamination was eliminated by DNaseI treatment (10U/sample) (Amersham Pharmacia Biotech), carried out at 37 °C for 30 min. Total RNA (5 μg) was reverse transcribed using 0.5 μg oligodT_(18)_, 0.5 mM dNTP mix, 5 mM DTT, 1× first strand buffer, 40U RNase Out, 200U SuperScript-III RnaseH Reverse Transcriptase (Life Technologies) in a final volume of 20 μl, following the manufacturer’s instructions. As negative control, total RNA not treated with reverse transcriptase was used. All qPCR assays were prepared in a final volume of 20 µl using 1 µl of diluted (1:5) cDNA, 10 µl of SYBR Green Master Mix (Bio-Rad Laboratories) and 0.5 µM sense and antisense specific primers. For each animal, analyses were carried out twice in duplicates using the Mastercycler CFX-96 (Bio-Rad Laboratories). Relative quantification for target genes was performed by the ^ΔΔ^Ct method^53^ using β-actin as reference gene. Temperature gradient and standard curves were performed to determine the optimal annealing temperatures and to assess primer efficiencies. Data were then reported as normalized fold expression (n.f.e.) ±SEM, over the value one arbitrarily assigned to one of the control animals at 17 PND.

### Protein extraction and Western blot analysis

Total proteins were extracted from rat testes in RIPA buffer as previously reported^52^. Proteins were resolved on 8% or 12% SDS-polyacrylamide gel electrophoresis and transferred to polyvinylidene fluoride filters (GE Healthcare) by TransBlot Turbo Transfer System (Bio-Rad Laboratories). Filters were treated with blocking solution (5% non-fat powdered milk, 0.25% Tween-20 in Tris-buffered saline, TBS, pH 7.6) for 3 h to prevent non-specific binding and then incubated with diluted primary antibodies (SIRT1 1:2000, Ac-p53^Lys370^ 1:500, p53 1:1000, Bax 1:500, Bcl_2_ 1:1000, catalase 1:2000, MnSOD 1:1000) in TBS-3% non-fat powdered milk solution overnight at 4 °C. Filters were washed in TBS-0.25% Tween 20, incubated with 1:1000 horseradish peroxidase-conjugated IgG (DAKO) in TBS-1% normal swine serum (NSS; DAKO) and then washed 3× in TBS-0.25% Tween-20. The immune complexes were detected using the ECL-Western blotting detection system (GE Healthcare). Filters were then stripped as previously reported^54^ and reprobed with anti-α tubulin antibody diluted 1:15000 to quantify protein content. Western blot signals were scanned and protein levels were plotted as quantitative densitometry analysis of signals. Data were expressed as target proteins/tubulin ratio ± SEM.

### Histological analysis

Testes were fixed in Bouin’s fluid and embedded in paraffin following standard procedures. Histological sections (5 µm) were deparaffinized with xylene, rehydrated with aqueous solutions of decreasing ethanol concentrations and used for immunohistochemistry or terminal deoxynucleotidyltransferase-mediated dUTP nick end labelling (TUNEL) assay. For frozen section preparation, the testes were covered with cryo-embedding medium OCT. After ensuring tissue was completely frozen, the tissue block was store at −80 °C, ready for sectioning (10 µm). Tissue sections were generated by using a Leica CM3050 S cryostat (Leica Microsystems).

### Fluorescence immunohistochemestry

Indirect immunofluorescence labelling and confocal microscopy were performed. SIRT1 expression and localization were investigated and its deacetylase activity on p53 was determined by Ac-p53^Lys370^ expression (antisera dilutions 1:100 and 1:50, respectively). BTB was identified by Cx43 and ZO-1 immunostaining (antisera dilution 1:100). To evaluate generation of ROS, frozen sections were incubated with the oxidative fluorescent dye DHE, as previously described^55^. Oxidative stress at DNA level was determined by 8-OHdG (antisera dilution 1:100) immunostaining. Nuclei were stained with DAPI or propidium iodide (Sigma-Aldrich). FITC and TRITC conjugated secondary antibodies were used. Sections were observed with a Zeiss LSM700 confocal microscope (Zeiss Italia, Italy).

### Antibodies

The antisera used for Western blot and immunohistochemistry were: mouse monoclonal anti-SIRT1 (ab110304) and rabbit monoclonal anti-p53 acetyl K370 (ab183544) from Abcam; mouse monoclonal anti-8-OHdG (12501) from QED Biosciences: rabbit polyclonal anti-γH2AX (A300-081A) from Bethyl Laboratories;rabbit polyclonal anti-p53 (#9282) from Cell Signaling Technology; rabbit polyclonal anti-Bax (sc-526) from Santa Cruz Biotechnology; rabbit polyclonal anti-ZO-1 (AB2272) and anti-MnSOD (06-984) from Merck Millipore; mouse monoclonal anti-Bcl_2_ (B9804),mouse monoclonal anti-catalase (C0979), rabbit polyclonal anti-CX43 (C6219) and mouse monoclonal anti-αtubulin (T6199) from Sigma-Aldrich; biotinylated goat anti-mouse IgG (IgG-B, sc-2039) from Santa Cruz Biotechnology; fluorescein isothiocyanate (FITC) and tetramethylrhodamine-5-(and 6)-isothiocyanate (TRITC) conjugated secondary antibodies from Jackson ImmunoResearch.

### Apoptosis detection

TUNEL assay was used to detect apoptosis by the ApoAlert DNA fragmentation kit according to manufacturer’s instructions (Clontech Laboratories). The extend of apoptotic death was determined in randomly selected tubules at 40× magnification and expressed as percentage of tubules with more than three apoptotic cells on total number of tubules.

### Statistics

Data are expressed as means ± SEM for BPA plasma levels, as means ± SEM for qPCR, Western blot and apoptotic index analyses. Statistically significant differences between groups were evaluated by the analysis of variance and the Tukey post-hoc test or by ANOVA followed by Duncan’s test for multi-group comparison.
